# Microindentation for In Vivo Measurement of Bone Tissue Mechanical Properties in Humans

**DOI:** 10.1002/jbmr.73

**Published:** 2010-02-23

**Authors:** Adolfo Diez-Perez, Roberto Güerri, Xavier Nogues, Enric Cáceres, Maria Jesus Peña, Leonardo Mellibovsky, Connor Randall, Daniel Bridges, James C Weaver, Alexander Proctor, Davis Brimer, Kurt J Koester, Robert O Ritchie, Paul K Hansma

**Affiliations:** 1 Hospital del Mar-IMIM-Universitat AutónomaBarcelona, Spain; 2 Department of Physics, University of CaliforniaSanta Barbara, CA, USA; 3 Coastal Marine BiolabsVentura, CA, USA; 4 Active Life Scientific, Inc.Santa Barbara, CA, USA; 5 Department of Materials Science and Engineering, University of CaliforniaBerkeley, CA, USA; 6 RETICEF, Instituto Carlos IIIMadrid, Spain

**Keywords:** bone, fracture, bone quality, instrument, clinical trials

## Abstract

Bone tissue mechanical properties are deemed a key component of bone strength, but their assessment requires invasive procedures. Here we validate a new instrument, a reference point indentation (RPI) instrument, for measuring these tissue properties in vivo. The RPI instrument performs bone microindentation testing (BMT) by inserting a probe assembly through the skin covering the tibia and, after displacing periosteum, applying 20 indentation cycles at 2 Hz each with a maximum force of 11 N. We assessed 27 women with osteoporosis-related fractures and 8 controls of comparable ages. Measured total indentation distance (46.0 ± 14 versus 31.7 ± 3.3 µm, *p* = .008) and indentation distance increase (18.1 ± 5.6 versus 12.3 ± 2.9 µm, *p* = .008) were significantly greater in fracture patients than in controls. Areas under the receiver operating characteristic (ROC) curve for the two measurements were 93.1% (95% confidence interval [CI] 83.1–100) and 90.3% (95% CI 73.2–100), respectively. Interobserver coefficient of variation ranged from 8.7% to 15.5%, and the procedure was well tolerated. In a separate study of cadaveric human bone samples (*n* = 5), crack growth toughness and indentation distance increase correlated (*r* = –0.9036, *p* = .018), and scanning electron microscope images of cracks induced by indentation and by experimental fractures were similar. We conclude that BMT, by inducing microscopic fractures, directly measures bone mechanical properties at the tissue level. The technique is feasible for use in clinics with good reproducibility. It discriminates precisely between patients with and without fragility fracture and may provide clinicians and researchers with a direct in vivo measurement of bone tissue resistance to fracture. © 2010 American Society for Bone and Mineral Research.

## Introduction

As people age, their bone strength deteriorates, and their bone becomes more susceptible to fracture.(1) The clinical consequence of this, the fracture, contributes to the morbidity and mortality of osteoporosis. Bone strength has been defined as the integration of bone mass and bone quality.(2) Available techniques for clinical estimation of bone strength or susceptibility to fracture are based mainly on bone mineral density (BMD) assessment(3) that can be reliably measured by densitometry techniques, but its sensitivity and specificity are modest.(3,4) Furthermore, its ability to predict the response to a treatment is limited, and only a small proportion of fracture risk reduction is explained by bone density increases.(5) Advanced bone imaging and analysis technologies promise better assessment of bone strength(6) but rely on potentially inaccurate assumptions about the tissue-level mechanical properties. The addition of other surrogates, such as biochemical markers, results in very limited improvement on these strength predictions.(7)

There is clinical and laboratory evidence that in addition to BMD, the mechanical properties of bone tissue may play a critical role in bone strength.(8–10) These mechanical properties would be expected to play a significant role in bone fracture risk, even though it has not been clear what mechanical properties are most important.(11–14) However, currently available methods for direct estimates of these properties require invasive bone sampling,(15) making routine use in clinics unfeasible.

Assessment of the intrinsic mechanical properties of bone tissue, as a key component of the widely used concept of bone quality, is limited. Besides the practical inconvenience of their routine measurement, the term *bone quality* is poorly defined and encompasses a series of geometric, microarchitectural, and tissue-composition elements.(15) As a consequence, the potentially relevant contribution of bone tissue strength to fracture risk in clinical practice cannot be evaluated, even though it is known that it deteriorates in osteoporosis and contributes to fracture propensity.(16)

Therefore, there is a critical need to better quantify bone mechanical properties at the tissue level, in particular, the ability of bone to resist the growth of cracks that result in bone fracture. This quantification is not only desirable for more complete clinical assessment of fracture risk but eventually also for treatment monitoring. Moreover, this development could help to better assess the effect of drugs on bone strength without the need for large and expensive prospective fracture trials.

Here we report the validation results of a novel microindentation technique capable of directly testing the mechanical endurance of bone tissue and suitable for a repeated measurement in patients. By measuring indentation distances, we assess the ability of bone to resist crack generation and propagation, the anatomic basis of fracture, in a series of women with osteoporosis-related fractures and controls. Moreover, we have performed exploratory studies on the anatomic substrate of the technique.

## Materials and Methods

### Subjects

This study involves 27 women with osteoporotic fractures (25 hip fractures and 2 multiple vertebral fractures) measured during the hospitalization following the event in the acute-care orthopedics ward and 8 controls of comparable age with no fractures from the Hospital del Mar, Barcelona, Spain. Fracture patients were excluded if there was some previous treatment with drugs for osteoporosis, corticosteroids use, a previous diagnosis of advanced renal or liver disease, neoplasia, malabsorption, thyroid or parathyroid disorder, immobilization, or inability to provide consent. Exclusion criteria for controls were identical, but in addition, control individuals were required to have no prevalent fracture. Thoracic and lumbar lateral radiographs validated the absence of subclinical vertebral fractures.

### Bone microindentation testing (BMT)

**Fig. 1:**
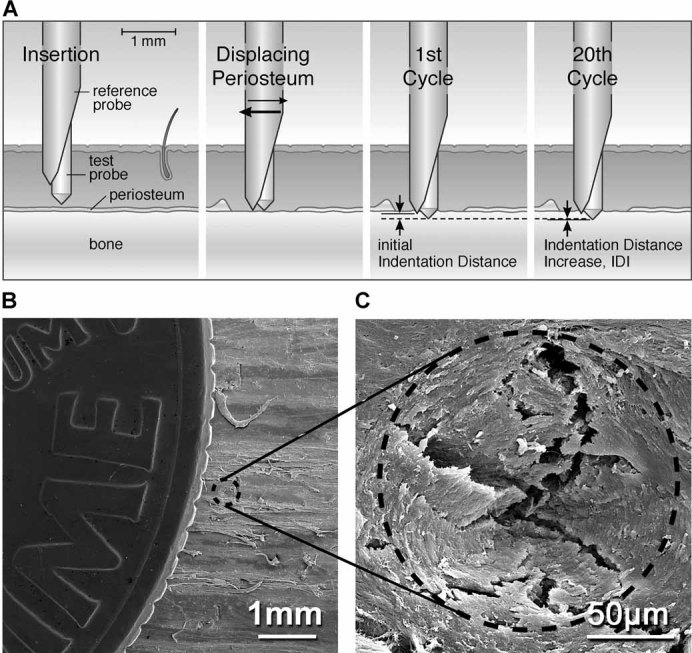
Indentation procedure for measuring material properties of bone in vivo and SEM imaging of an indent on a human bone sample. (*A*) Illustration of the method for obtaining indentation measurements, including insertion of the test probe assembly, displacing the periosteum with the reference probe, first-cycle indentation, and last-cycle indentation, which determines the IDI with respect to the first cycle. (*B*) SEM image of an indentation (*encircled by dashed line*) being compared to a dime (the smallest U.S. coin). (*C*) This magnified SEM image of the indentation shows microcracks created during the repetitive loading cycles at a constant force.

The reference point indentation (RPI) instrument (which was called the *tissue diagnostic instrument*(17) and the *bone diagnostic instrument*(18–20) in previous publications) can measure bone mechanical properties, in particular, the resistance to fracture, at the tissue level (Fig. 1*A*). The complete BMT protocol involves 10 steps: (1) Attach a presterilized, disposable probe assembly to the head unit of the RPI instrument.(17) (2) Apply alcohol and local anesthesia to the testing site (midshaft of anterior tibia). (3) Use the guidance arm with the vertical slider to position the head unit over the midshaft anterior tibia. The head unit must be perpendicular to bone's surface within about 15 degrees. Since the head unit is held vertical by the guidance arm with the vertical slider, this is achieved by holding the patient's foot and leg such that the midshaft of the anterior tibia is level to within an estimated 15 degrees or less. (4) Holding the sterile probe assembly with a sterile glove, lower head unit vertically along slider to insert the probe assembly through the skin to rest on the bone surface. (5) Displace the periosteum from the measurement area by moving the reference probe by hand laterally along the surface of the bone a distance of approximately 5 mm for a series of five times, and then place it in the center of this approximately 5-mm region for measurement. (6) Release the probe assembly so that it rests with the full weight of the head unit on the bone. (7) Actuate the measurement cycle, which first removes an initial 2.5-N force on the test probe (used to keep the test probe from sliding back into the reference probe during insertion) and then begins a series of precycles at 4 Hz that incrementally increase up to a threshold force of order 2.5 N and then runs the 20 indentation cycles at 2 Hz each with a maximum force of 11 N. (8) Repeat steps 3 through 7 to obtain measurements at five or more locations. Each measurement location should be separated by at least 2 mm from other measurement locations. (9) After the final measurement, raise the head unit away from tibia, and detach and discard the disposable probe assembly. (10) Wipe the measurement site with alcohol, and apply a bandage. Local edema or advanced skin disorder and infection in the measurement area would have precluded use of this technique. Warfarin treatment or severe coagulation defects have to be considered for careful local hemostasis.

The indentations are small, on the order of 375 µm across (Fig. 1*B*), so they are not harmful to the patient. They are large enough, however, that the bone is fractured (Fig. 1*C*) as the test probe indents the bone. The more easily the bone is fractured, the farther the test probe will indent the bone. Thus we quantify the bone fracture resistance by measuring the indentation distances achieved in a measurement. The indentation has to be performed by the test probe perpendicular to the bone surface, with a tolerance of ±15 degrees to obtain reliable results.

**Fig. 2:**
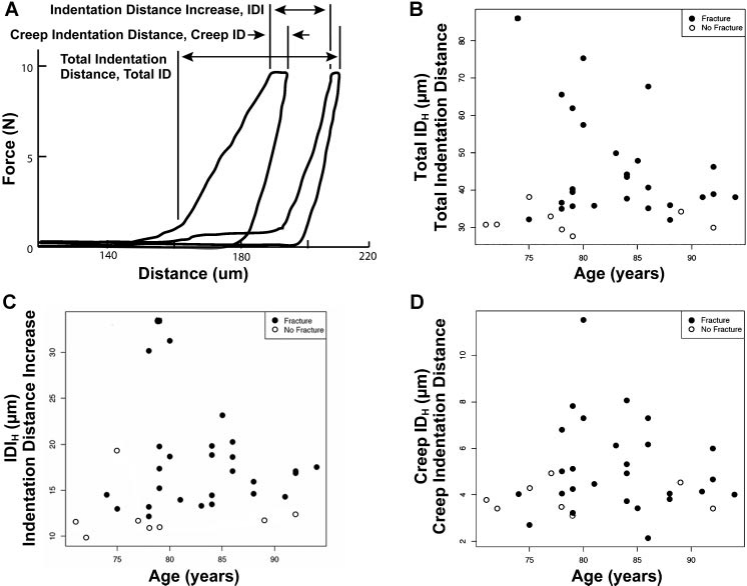
Parameters are calculated from force-versus-distance data obtained by the RPI instrument. The parameters include indentation distance increase (IDI), total indentation distance (total ID), and creep indentation distance (creep ID) measured in the first cycle. (*A*) The IDI is defined as the increase in the indentation distance in the last cycle relative to the indentation distance in the first cycle (see Fig. 1*A*). The creep ID is determined by the increase in distance while the force is held constant at the maximum value for a duration of one-third of the first indentation cycle. The total ID is defined as the total distance the test probe is inserted into the bone from touchdown to the end of the twentieth cycle. (*B–D*) Results from clinical trials of each parameter with fracture (*n* = 27) and control (*n* = 8) patients. Note that fracture patients usually had higher indentation distances. The subscript *H* on the graphs indicates that the parameters were measured with the Hospital del Mar protocol. This is important because the values of these parameters depend on the measurement protocol.

The control system for the reference point indentation instrument supplies a modified triangular wave to its internal force generator for the 20 indentation cycles used in measurements. The modified triangular waveform consists of one-third of a cycle of linear increase, followed by one-third of a cycle hold at maximum force (for measuring creep), and then one-third of a cycle of linear decrease. The total cycle time is 500 ms. The purpose of the hold at maximum force is to monitor creep effects and to minimize the effect of the remaining creep during the linear decrease. After the cycles are complete, a computer displays the first and last (twentieth) force-versus-distance curves (Fig. 2*A*). Three indentation parameters are defined in the figure.

Total time for the test is 10 minutes. The patient experiences minimal discomfort (only during the local anesthesia injection), and no complications have been observed whatsoever.

### DXA measurement of BMD

BMD with dual-energy X-ray absorptiometry (DXA) using a Hologic QDR 4500 SR Bone Densitometer (Hologic, Inc., Waltham, MA, USA) was measured at the nonfractured hip within 4 weeks of admission in a subset of 14 individuals randomly chosen (nine fracture cases and five controls) from our clinical cohort.

### Statistical analysis

Normality of continuous variables was assessed by Q-Q plots. Analysis of covariance was used to obtain and compare age-adjusted means. Pearson correlation index was computed to assess the relationship between continuous variables. The ability of the indentation distance parameters to discriminate between those who have a fracture and those who do not was assessed by calculating the area under the receiver operating characteristic (ROC) curve.

### Preclinical experiments on cadaveric bone

To connect indentation distance increase (IDI), as determined by the reference point indentation instrument, to a conventional measure of fracture resistance on machined samples, we measured both IDI and crack growth toughness on cadaveric bone samples from a group of five donors (aged 17 to 74 years). This is a totally different group from the clinical group discussed earlier. There were eight samples, three for the 74-year-old male, two for the 23-year-old male, and one for each of the other three subjects that gave crack growth data. In the case of the multiple measurements on one donor, the multiple measurements were averaged together to give one data point for the correlation calculation. For IDI data, there were 15 samples, 3 for each donor and 10 tests on each sample for a total of 150 measurements. Again, all measurements on one donor were averaged together to give one data point for the correlation calculation. We were able to do more measurements for the IDI because we could do multiple measurements on each sample, and no special machining was required. The samples were cut from the tibia with dimensions of the order of 2 cm in length and width and the full thickness of the cortical bone. The bone samples were stored in a −80°C freezer. Prior to testing, the samples were brought to room temperature, gently stripped of soft tissue, and placed in Hank's balanced saline physiologic buffer solution(21) to ensure hydration. The surface of the bone was not polished. Figure 1*C* shows the microcracks opened by the indentations. Microcracks are opened during RPI testing just as cracks are opened on machined samples during *R*-curve testing. Thus it is reasonable to compare the results of RPI testing with the crack growth toughness from *R*-curve testing.

Indentation testing was conducted by the RPI instrument. The bone samples were held in a vice submerged in physiologic buffer and tested under the buffer. The indentations were normal to the outside surface of the cortical shell. Each sample had a minimum of 10 tests conducted in varying locations. Three samples were tested from each donor. Each individual test was analyzed by software that was written to compute a variety of mechanical parameters such as IDI. The second method used crack resistance curves (*R* curves) to determine the crack growth toughness. Compact tension samples were sectioned and notched transverse to the bones' long axis. The notch orientation was such that the nominal crack growth direction was transverse to the long axis of the tibia. We used nonlinear elastic fracture mechanics testing of the bone samples under hydrated conditions in situ in an environmental scanning electron microscope (ESEM) to permit resistance curve measurements for growing short cracks in the transverse orientation less than 1000 µm in size. Additional details on the testing method and procedure used in this preclinical experiment are discussed by Koester and colleagues.(22) The stress intensity *K* and crack extension data were linearly extrapolated to determine the growth toughness Δ*K*/Δ*a* (MPa√m/µm), which is obtained from the slope of the *R* curve.(22–24) Higher growth toughness signifies a bone that is less prone to continued crack propagation.

## Results

### BMT clinical experiment

**Fig. 3:**
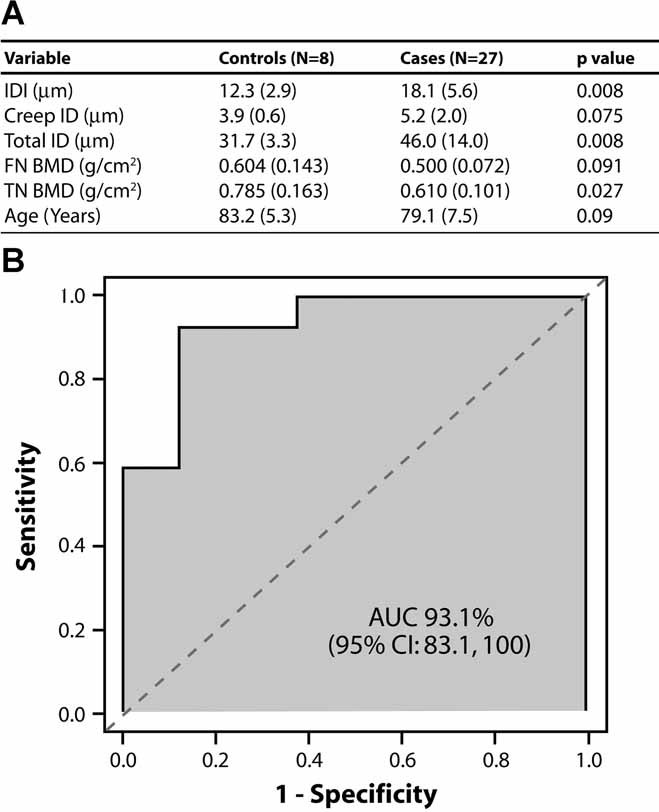
Data results including statistics and a receiver operating characteristic (ROC) curve. (*A*) Age-adjusted statistical results for IDI (µm), creep ID (µm), total ID (µm), femoral neck bone mineral density (FN BMD, g/cm^2^), and total-hip bone mineral density (TH BMD, g/cm^2^). (*B*) The ROC curve displays the clinical results from Hospital Del Mar, Barcelona. The area under the curve (*AUC*) is a scalar quantity to gauge the performance of the curve. An *AUC* of 100% would represent a perfect model; however, an area going along the line of discrimination (*dashed diagonal*) would be a completely random model.

Two of the three measured indentation parameters are significantly greater for patients with fractures than for control patients (Figs. 2 and 3). Note also that there is no apparent correlation between age and indentation values, at least in the small population of elderly women investigated in this study (Fig. 2). The ROC curve shows that the total indentation distance (total ID) is a good discriminator between patients with and without fractures.(25) The area under the ROC curve (*AUC*) value(26) in this study for total ID was of 0.931 [95% confidence interval (CI) 83.1–100], 90.3% for IDI (95% CI 73.2–100), and 73.6% for creep ID (95% CI 56.4–90.9).

Interobserver variability was assessed by separated measurements performed by two observers in 14 individuals. The coefficient of variation ranged from 8.7% (for IDI) to 15.5% (for total ID).

Differences between cases and controls are shown in Fig. 3*A*. As expected, BMD differences were observed. However, the correlation between total-hip BMD and IDI (*r*^2^ = −0.127, *p* = .211) and total ID (*r*^2^ = −0.264, *p* = .06) was low, indicating, as might be expected, that measurements of bone loss (DXA) alone cannot predict bone tissue mechanical properties as measured by the RPI instrument.(25)

### Preclinical experiments on cadaveric bone

The results for the comparison between IDI and crack growth toughness are shown in Table 1. The IDI is much greater for the 74-year-old male subject with an IDI of 20.49 ± 6.88 µm, whereas it is very low for younger subjects. We measured the IDI of cadaveric bone from additional older subjects but were unable to generate an *R* curve for each of the subjects because of the geometry of the bones and the requirements of our testing method.(19) For example, with most of the older individuals who had osteoporosis, there was very little cortical shell to work with on the limited number of samples we had available. Since we had only one older subject from whom we got multiple tests, our results can only be regarded as preliminary. Future testing to compare IDI and crack growth toughness on a wider range of individuals would be valuable. This may require novel methods for determining crack growth toughness.

**Table 1:** Indentation Distance Increase and Crack Growth Toughness for Each Donor Sample Tested for Correlations

Age/sex	IDI ± SD (µm) (N)	Δ*K*/Δ*a* (MPa√m/µm) (N)
74/M	20.49 ± 6.88 (3)	0.0365 (3)
23/M	14.75 ± 3.12 (3)	0.0428 (2)
17/F	13.97 ± 2.76 (3)	0.0405 (1)
44/F	12.89 ± 3.70 (3)	0.0426 (1)
22/F	12.43 ± 2.49 (3)	0.0455 (1)

*Note:* The number of samples tested from each donor *n* for each test is shown next to the test result in parentheses. Note the inverse relationship between IDI and Δ*K*/Δ*a* because high IDI and low Δ*K*/Δ*a* correspond to a high fracture risk.

**Fig. 4:**
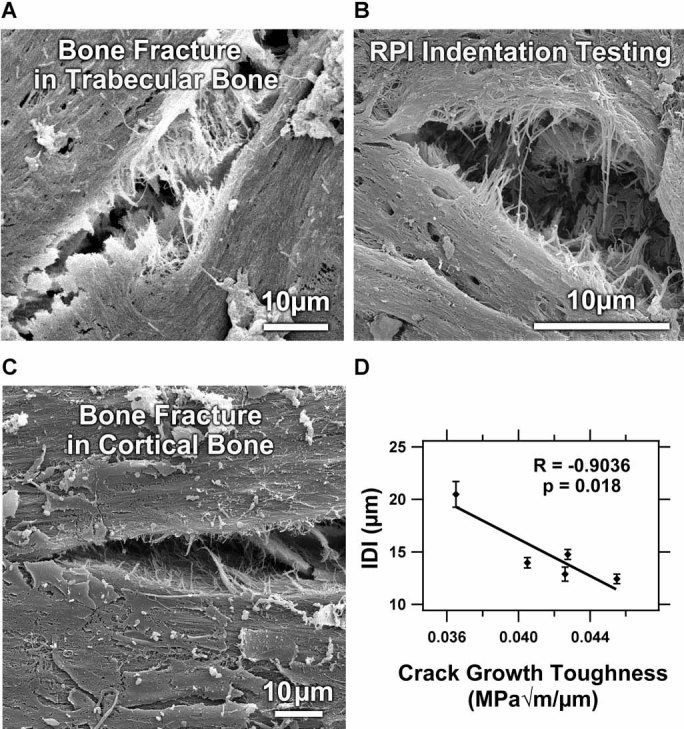
SEM images of cadaveric human bone samples that were fractured and exhibit crack bridging, which resists crack extension. The crack growth toughness of samples was compared with the indentation distance increase (IDI). (*A*–*C*) The samples in panels *A* and *C* were fractured in fluid,(27) and microcracks were observed, whereas the sample in panel *B* displays a microcrack created by the RPI instrument during repetitive indentations. It resembles the microcracks in both *A* and *C*. (*D*) Comparison between IDI and crack growth toughness(22) (slope of *R* curve) obtained for samples from five donors. High IDI and low crack growth toughness are associated with bones that are prone to fracture. The graph shows this trend by relating high IDI to low crack growth toughness and vice versa. The linear fit has a Pearson correlation of −0.904, with *p* = .018 (one-tailed) and *p* = .035 (two-tailed). We believe that the one-tailed test is justified because we anticipated the direction of the trend: High IDI corresponds to low crack growth toughness. Because of the limited number of samples and subjects, this correlation should be regarded as preliminary until a more complete investigation is done.

Figure 4*A–C* shows scanning electron microscope (SEM) images of human bone samples that were fractured and exhibit crack bridging, which resists crack extension. The crack growth toughness of the samples then was compared to the IDI. In samples fractured in fluid,(27) microcracks were observed by SEM, and their appearance was similar to microcracks created by the RPI instrument during repetitive indentations. Comparisons between IDI and the crack growth toughness(22) (slope of the *R* curve) for samples from five donors showed that high IDI and low crack growth toughness are associated with bones that are prone to fracture. The graph shows this trend by relating high IDI to low crack growth toughness and vice versa. Pearson's correlation coefficient between the IDI and crack growth toughness is −0.9036, with *p* = .018 (Fig. 4*D*). The coefficient is negative owing to the inverse relationship between IDI and crack growth toughness.

## Discussion

Here we describe the validation study of a novel device that performs bone microindentation testing (BMT) of bone in vivo in a series of patients with and without osteoporotic fractures. BMT discriminates between cases and controls and measures parameters different from BMD. Preclinical studies in human cadavers suggest that BMT induces separation of mineralized collagen fibrils and initiation of cracks, very likely the basic mechanism of fracture, thus directly measuring the mechanical competence of bone tissue to resist fracture.

The validation process has followed the usual sequence of developing a suitable measurement protocol and validating the ability of the technique to discriminate between cases with and without the studied condition. Developing the clinical protocol herein described covered the first objective. The anterior midshaft of the tibia was chosen for the measurements owing to easy accessibility, as well as also offering a relatively flat surface where the indentation could be made almost perpendicular to the surface. Periosteum is displaced to avoid interference with the measurements. Interobserver variability also was assessed and resulted in acceptable values that make feasible cross-sectional interindividual as well as longitudinal within-individual comparisons.

The ability to discriminate between cases with and without fracture was demonstrated by the finding of differences in indentation distances between cases and controls. Total ID and IDI showed significant differences, whereas for creep ID, although there was a trend, the difference did not reach significance, very likely owing to the lesser magnitude of this measurement. To further explore this, although the number of cases is limited, the areas under the ROC curve were calculated, yielding excellent values (above 90%) for the two indentation parameters total ID and IDI.

When the BMT values were compared with densitometry measurements in a subset of cases, the differences appeared to be more significant for the former, and the *AUC* values for BMT also were well above the best described for densitometry, even in combined sophisticated assessments.(3–4) Furthermore, there was no significant correlation between the two, further stressing the fact that different parameters of bone properties were studied.

Therefore, tissue mechanical properties, in particular, the resistance to fracture, as quantified by the total ID and the IDI, were significantly different between patients with and without fractures in the clinical results presented here. These clinical results are consistent with six previous laboratory case-control studies in which more easily fractured bone was found to have greater IDI values.(17–20,28) These results can, at least in part, be understood from comparisons of the local microstructure of the cracks opened by the RPI instrument and the cracks involved in bone fracture (Fig. 4). From this study, it appears that as the resistance to crack extension decreases and IDI increases, the probability of fracture increases.

Many possible mechanisms exist that can change the tissue mechanical properties of bone.(13–14,29) These include microcracking(30) and microdamage,(31) changes in mineralization,(12) changes in mineral crystal size,(32) changes in the organic matrix,(33) including posttranslational changes in collagen,(34) changes in collagen fibril orientation,(35–37) and changes in noncollagenous proteins.(38,39) Clinical conditions such as osteogenesis imperfecta further demonstrate the importance of tissue mechanical properties on bone fracture risk. Until now, however, it has been impractical to measure bone material properties in living patients without removing bone samples.

Bone fracture in both trabecular and cortical bone begins with the separation of mineralized collagen fibrils and the initiation of cracks,(38,40–44) as depicted in the SEM images from our laboratory experiments.(27,42,45) The RPI instrument opens cracks that are very similar to those observed following bone fracture. The resistance to extension of the cracks can be quantified, on machined specimens, by resistance-curve (*R*-curve) analysis of the slope of a plot of stress intensity versus crack extension as first shown by Vashishth.(22,24) The slope of the *R* curve is called the *crack growth toughness*, and the larger the crack growth toughness, the larger is the resistance to the extension of cracks. We thus would expect an inverse relationship with IDI, which is smaller if there is more resistance to the extension of the cracks under the tip, as seen in our experiments. This is indeed the case, as demonstrated by the significant negative correlation. This significant correlation relates IDI to crack growth toughness and provides a greater understanding of the physical significance of IDI. This shows that repetitive indentation normal to the bone, as used to determine IDI, is very similar to crack growth toughness; however, IDI can be determined in vivo, whereas crack growth toughness cannot.

There is a substantial history of atomic force microscopy and indentation measurements on bone. A recent review(46) discusses 149 papers. Most commonly, elastic modulus and hardness are measured. Since, however, it is not clear what material parameter (or combination of parameters) best correlates with fracture risk,(11–14) we measured a large number of parameters, including elastic modulus, hardness, initial indentation distance, total indentation distance, indentation distance increase, creep, energy dissipation, and others.(19) From these studies, we discovered that elastic modulus and hardness did not distinguish the bone of patients with and without fractures as well as the parameters reported here, which involved not just one indentation cycle but 20 cycles. It was unclear initially why hardness was a poor indicator of fracture compared with the first-cycle indentation distance because for our tip geometry, a 90-degree cone, hardness is simply the maximum force divided by π times the first-cycle indentation distance squared. The problem was discovered to result from the combined effect of outliers with small indentation distances. They inflated and dominated averages once the raw data of the indentation distance were inverted and squared. Elastic modulus suffered from the same problem, but to a lesser extent. Since elastic modulus depends on the unloading slope after the indentation is made, we were measuring the “elastic modulus” of cracked material, which would not be expected to be characteristic of the uncracked material.

Our BMT technique differs substantially from the previously described osteopenetrometer in several aspects. The osteopenetrometer(47) was developed for intraoperative measurement of bone strength. It used a much larger indenter, over 2 mm in diameter, that indented trabecular bone by distances on the order of 10 mm at forces of hundreds of newtons. These large distances were necessary to average over many trabeculae. Thus the osteopenetrometer is very different from the RPI instrument, which makes microscopic indentations in cortical bone without surgically exposing the bone. The key advance of BMT over previous indentation studies is that the RPI instrument allowed indentation measurements on the bone of living patients without surgically exposing the bone or removing the bone from the patient.

Our study has some limitations. Although we might assume homogeneous mechanical properties of the bone tissue volume unit, our measurements are limited to a cortical compartment and in a given bone, the tibia. Whether this is fully representative of other bones remains speculative at this point, although the primary resistance to mineralized collagen fibril separation might be assumed to be similar across all different skeletal compartments and regions. The number of cases studied is limited, although the differences between cases and controls were strongly significant, which makes a chance finding highly unlikely. Also, our experience is limited to a single center and to a precise group of patients, elderly postmenopausal women. Replication in other groups and populations is warranted.

In summary, we report a novel technique suitable for in vivo measurement of bone tissue strength in a clinical setting. The technique is based on creating microfractures and measuring the overall resistance of bone to the propagation of these microfractures. This represents a direct assessment of bone tissue mechanical strength in patients, an important component of the properties encompassed under the umbrella of “bone quality.” Although more research will be needed to use IDI and other parameters measured by the RPI instrument to quantify the contribution of tissue mechanical properties to bone fracture risk, it is already possible to use these parameters to inform the development of novel therapies. This research also opens the possibility of investigations into the differences in the nanoscale fracture mechanisms between bones with different values of IDI.
